# Automated dose dispensing service for primary healthcare patients: a systematic review

**DOI:** 10.1186/2046-4053-2-1

**Published:** 2013-01-08

**Authors:** Juha Sinnemäki, Sinikka Sihvo, Jaana Isojärvi, Marja Blom, Marja Airaksinen, Antti Mäntylä

**Affiliations:** 1 Assessment of Pharmacotherapies, Finnish Medicines Agency, P.O. Box 55, 00034 FIMEA, Kuopio, Finland; 2 Division of Social Pharmacy, Faculty of Pharmacy, University of Helsinki, P.O. Box 56, 00014 University of Helsinki, Helsinki, Finland; 3 Finohta (Finnish Office for Health Technology Assessment), National Institute for Health and Welfare, P.O. Box 30,, 00271, Helsinki, Finland

**Keywords:** Automated dose dispensing, Medication safety, Primary healthcare, Patient safety, Medication use

## Abstract

**Background:**

An automated dose dispensing (ADD) service has been implemented in primary healthcare in some European countries. In this service, regularly used medicines are machine-packed into unit-dose bags for each time of administration. The aim of this study is to review the evidence for ADD’s influence on the appropriateness of medication use, medication safety, and costs in primary healthcare.

**Methods:**

A literature search was performed in April 2012 in the most relevant databases (*n* = 10), including the Medline, Embase, and Cochrane Library. The reference lists of the studies selected were manually searched. A study was included in the review if the study was conducted in primary healthcare or nursing home settings and medicines were dispensed in unit-dose bags.

**Results:**

Out of 328 abstracts, seven studies met the inclusion and reporting quality criteria, but none applied a randomized controlled study design. Of the four controlled studies, one was a national register-based study. It showed that the patient group in the ADD scheme more often used three or more psychotropic drugs and anticholinergics than patients using the standard dispensing procedure, while women in the ADD group used less long-acting benzodiazepines and both genders had fewer drug-drug interactions. In another, regional controlled study, the ADD group consisted of patients with higher risk of inappropriate drug use, according to all indicators applied. The third controlled study indicated that ADD user drug treatments were more likely to remain unchanged than in patients using a standard dispensing procedure. A controlled study from Norway showed that ADD reduced discrepancies in the documentation of patient medication records. Costs were not investigated in any of the studies.

**Conclusions:**

A very limited number of controlled studies have explored ADD in primary healthcare. Consequently, the evidence for ADD’s influence on appropriateness and safety of medication use is limited and lacking in information on costs. The findings of this review suggest that patients using the ADD have more inappropriate drugs in their regimens, and that ADD may improve medication safety in terms of reducing the discrepancies in medication records. Further evidence is needed to draw sound conclusions on ADD’s outcomes.

## Background

Medication errors are preventable events that may cause or lead to inappropriate medication use [1]. They are common and can occur at any stage of the medication process in inpatient and outpatient care [2,3]. Therefore, it is important to identify the weak stages of the medication process and develop preventive mechanisms to avoid the errors. Automated dose dispensing (ADD) may serve to enhance medication safety, particularly among elderly outpatients with multiple medications.

ADD is a service in which regularly used medicines are machine-packed into unit-dose bags for each time of administration [4]. ADD is used in primary healthcare in Sweden, Denmark, Norway, and Finland, Sweden having the highest volume with 185,000 patients using ADD in 2009 [4-8]. Community pharmacies in Denmark have been obliged to provide ADD since 2003 [6]. The medicine agencies of Sweden and Norway established guidelines on dose dispensing in 2010 and 2000, respectively [9,10].

In Finland, ADD was first launched in 2002, [4] and implemented through legislation in 2011 [11]. The service is delivered nationally through community pharmacies that buy the dose bags from two providers [12]. Currently, about 300 out of a total of 600 community pharmacies provide the ADD service. The number of patients using the scheme is about 20,000.

The Ministry of Social Affairs and Health has recommended the ADD service for elderly primary healthcare patients to ensure safe medication [13]. Since 2006, the ADD service has been partly reimbursed by the public insurance that covers the entire population [14]. The service is only reimbursed for aged patients (≥75 years) using six or more reimbursable prescription medicines that are suitable for ADD.

The ADD service is expected to enhance patient safety, decrease medication costs, and save nurses working time in the primary healthcare [4]. No published systematic reviews have been conducted to evaluate the outcomes of the ADD service. The aim of this study was to review the evidence for influence of ADD on the appropriateness of medication use, medication safety, and costs in primary healthcare.

## Methods

### Literature search

A literature search was performed in April 2012 on the following databases: Medline, Medline in-process, and other non-indexed citations, Cochrane database of systematic reviews, Cochrane central register of controlled trials, Cinahl, Journals@Ovid, NHS Economic evaluation database (EED), Health technology assessment database (HTA), Database of abstracts of reviews of effectiveness (DARE), and Embase. Key search terms included: automated medication/drug dispensing, automated medication/drug distribution, automated dose dispensing/distribution, automated dispensing system, multidose drug dispensing/distribution, and unit-dose dispensing/distribution. Reports and studies published from early 1995 to April 2012 were included in the literature search. The search was not limited by language. An example of the search strategy is in Table 1. The reference lists of the studies selected were manually searched. Finnish literature databases were also searched, using a strategy similar strategy to that of the international databases.

**Table 1:** Search strategy for the medline

**Ovid MEDLINE(R)**	
1	automated medication dispens*.ti,ab. (20)
2	automated medication distribut*.ti,ab. (6)
3	automated drug distribut*.ti,ab. (5)
4	automated drug dispens*.ti,ab. (14)
5	automated dose-dispens*.ti,ab. (3)
6	automated dose distribut*.ti,ab. (0)
7	automated dispensing system*.ti,ab. (29)
8	multidose drug dispens*.ti,ab. (0)
9	multi-dose drug dispens*.ti,ab. (2)
10	multidose drug distribut*.ti,ab. (1)
11	multi-dose drug distribut*.ti,ab. (1)
12	unit-dose dispens*.ti,ab. (45)
13	unit-dose distribut*.ti,ab. (33)
14	(automat* adj2 (dispens* or distribut*) adj2 (device* or system* or scheme*)).ti,ab. (96)
15	(automat* adj2 dose dispens*).ti,ab. (7)
16	(automat* adj2 dose distribut*).ti,ab. (10)
17	((multidose or multi-dose) adj2 dispens*).ti,ab. (8)
18	((multidose or multi-dose) adj2 distribut*).ti,ab. (5)
19	(unit-dose adj2 (dispens* or distribut*)).ti,ab. (218)
20	or/1-19 (350)
21	(news or letter or comment or editorial or interview or historical article).pt. (1438428)
22	20 not 21 (338)
23	limit 22 to yr=”1995-current

### Inclusion and exclusion criteria

A study was included in the review if it was conducted in primary healthcare or nursing home settings and the medicines were dispensed for patients in unit-dose bags. Studies performed in hospital settings were excluded, as well as those with manually distributed medicines to unit-dose cups or any similar procedures. Control groups were not required, because there were few studies performed on ADD in primary healthcare. Qualitative studies and case reports were excluded. Studies applying outcome measures that were associated with the appropriateness of medication use or medication safety were included. Studies regarding costs or any other type of economic evaluation of ADD were also included. In short, the following PICO was applied in this study: Patients (patients from primary healthcare or nursing homes), Intervention (ADD), Comparison (usual care/not ADD; not required), and Outcomes (appropriateness of medication use, medication safety, and costs).

### Data extraction

Two reviewers (JS, SS) independently selected studies, based on abstracts according to inclusion and exclusion criteria. Disagreements were resolved through discussion and consensus. Study characteristics, aim of the study, description of ADD, study population and data collection, outcome measures, and main results categorized to appropriateness of medication use, medication safety, and costs were extracted by one of the authors (JS) to a table (Table 2). Table 2 was carefully reviewed by the other authors.

**Table 2:** Description and results of the studies on automated dose dispensing (ADD) in primary healthcare

**Reference, country, and study design**	**Aim of the study**	**Description of automated dose dispensing (ADD) according to article’s text**^**a**^	**Population and data collection**	**Outcome measures**	**Outcome specification and main results**
**Controlled studies**					
Sjöberg et al. [15], 2012, Sweden	To compare changes in drug treatments within and outside ADD	Level 2	154 community-dwelling or nursing home residents ≥65 years of age (patients using ADD n = 107, not using ADD n = 47). Data on drug treatments were extracted from the medical records (t = 0 months) and from the SPDR (t = 6 months). A multi-level analysis was performed, with drugs at the first level and individuals at the second.	Number of changed (withdrawn, dosage adjusted, or newly prescribed) and not changed drugs.	*Appropriateness of medication use*
Controlled register study					The risk of medication to be classified as unchanged was higher among ADD users (OR 1.66, 95% CI 1.20-2.31, adjusted for age, sex, cognition, year of data collection, subgroup of drug).
Sjöberg et al. [16], 2011, Sweden	To investigate association between ADD and quality of drug treatment	Level 3	All community-dwelling or nursing home residents from Västra Götaland ≥65 years of age in late 2007 and having ≥2 health care visits and ≥2 diagnosis in 2005–2007. Study group: ADD users (n = 4927). Control group: patients not using ADD (n = 19 219). Data were collected from the SPDR in 2007 linked with register data on patient diagnoses and residence.	Five quality indicators for potential IDU:	*Appropriateness of medication use*
Controlled cross-sectional register study				1. Use of ≥10 drugs	ADD users had a higher prevalence of all indicators of potential IDU (5.9-55.1%) than the control population (2.6-4.9%) (*P* <0.0001). After adjustment for age, sex, burden of disease, and residence, risk of all indicators of potential IDU were higher among ADD users (ORs 1.36-5.48; 95% CI 1.18-6.30).
				2. Use of long-acting benzodiazepines	
				3. Use of anticholinergic drugs	
				4. Use of ≥3 psychotropic drugs	
				5. Potential DDIs	
Wekre et al. [17], 2010, Norway	Impact of ADD on inconsistencies in medication records between GPs and home care services	Level 3	A convenience sample of 59 patients. Medication records were collected 0.5 years before and 1 year after the ADD implementation.	Number of discrepancies between the patients’ medication records at the GPs and at the home care services	*Medication safety*
					ADD did not change the number of medication records with discrepancies (before 47 and after 45 out of 59, *P* = 0.774, n.s.), but reduced total number of discrepancies by 34% (*P* < 0.001).
Controlled before-after study					
Johnell and Fastbom [18], 2008, Sweden	Whether the use of ADD is associated with potential IDU	Level 2	All Swedes ≥75 years of age who were registered in SPDR. Study group: ADD users (n = 122 413). Control group: patients not using ADD (n = 608, 692). Data were collected from the SPDR in 2005.	Four quality indicators for potential IDU:	*Appropriateness of medication use*
					ADD users had a higher prevalence of all indicators of potential IDU (8.8-22.1%) than the control population (2.4-4.9%).
Controlled cross-sectional register study				1. use of long-acting benzodiazepines	
				2. use of anticholinergic drugs	After adjustment for age and number of dispensed drugs, risk of using any IDU, anticholinergic drugs and ≥3 psychotropic drugs were higher among ADD users (ORs 1.43-4.93; 95% CI 1.40-5.17). Contrasting relationship prevailed for long-acting benzodiazepines among women and potentially serious DDIs among women and men (ORs 0.69-0.80; 95% CI 0.66-0.83).
				3. use of ≥3 psychotropic drugs	
				4. potential DDIs	
**Uncontrolled studies**					
Olsson et al. [19], 2010, Sweden	Extent and quality of drug prescribing in younger elderly (65–79 years) and older elderly (≥80 years) receiving ADD	ADD is mentioned but no description is given.	All residents of nursing homes and dementia special care units ≥65 years of age (n = 3705) from the County of Jönköping. Data on prescribed drugs were collected from the national pharmacy drug register.	Five quality indicators for potential IDU:	*Appropriateness of medication use*
Cross-sectional register study				1. Use of long-acting benzodiazepines	Influence of ADD on potential IDU not studied. Potential IDU prevalences ranged from 7.6% to 41.2%. Prevalences of potential IDU were mainly higher among younger (65–79 years) than older (≥80 years) residents (not statistically tested).
				2. Use of anticholinergic drugs	
				3. drug duplications	
				4. Use of ≥3 psychotropic drugs	
				5. Potential DDIs	
van den Bemt et al. [20], 2009, the Netherlands	Frequency of medication administration errors and potential risk factors for these errors in nursing homes using ADD	Level 2	In all, 2025 administrations to 127 residents of three nursing homes were observed by one pharmacy technician.	Medication administration error rates	*Medication safety*
					Administration error rate for all administered medications observed (via ADD and without ADD) was 21.2% (n = 428 errors). Most common error type was wrong administration technique (n = 312). The risk for administration errors was higher when medicine was not supplied by ADD (OR 2.92; 95% CI 2.04-4.18).
Prospective observational study					
Bergman et al. [21], 2007, Sweden	Quality of drug therapy among nursing home residents using ADD	Level 1	All nursing home residents ≥65 years of age (n = 7904) from Gothenburg area. Data were collected from the Swedish national drug register for ADD users.	Five quality indicators for potential IDU:	*Appropriateness of medication use*
Cross-sectional register study				1. use of long-acting benzodiazepines	Influence of ADD on potential IDU not studied. Potential IDU prevalences ranged from 12.1% to 45.2%. The proportion of potential IDU was higher among 65–79 year-old residents than those ≥80 years old (*P* 0.001-0.015).
				2. Use of anticholinergic drugs	
				3. Drug duplications	
				4. Use of ≥3 psychotropic drugs	
				5. Potential DDIs	

ADD, Automated dose dispensing; DDI, Drug-drug interaction; GP, General practitioner; IDU, Inappropriate drug use; n.s., Not significant; SPDR, Swedish Prescribed Drug Register.

^**a**^**Levels of description:** Level 1: Drugs are machine-packed into unit-dose bags. One unit-dose bag contains all the tablets that are administered to a patient at the same time; Level 2: In addition to level 1, each bag has a label with the following information: patient’s name, the name(s) of the medication(s), and the date and time of administration; Level 3: In addition to levels 1 and 2, a medication record was set up.

### Quality assessment of the studies

The quality of reporting of the studies selected was assessed using the STROBE checklist (Additional file 1) [22]. The proportion of adequately reported items (yes) to applicable questions was counted. The quality was considered good when the proportion of adequately reported items (yes) to applicable questions was higher than 80% and acceptable when the proportion was >60% but <80%.

## Results

### Included studies

**Figure 1:**
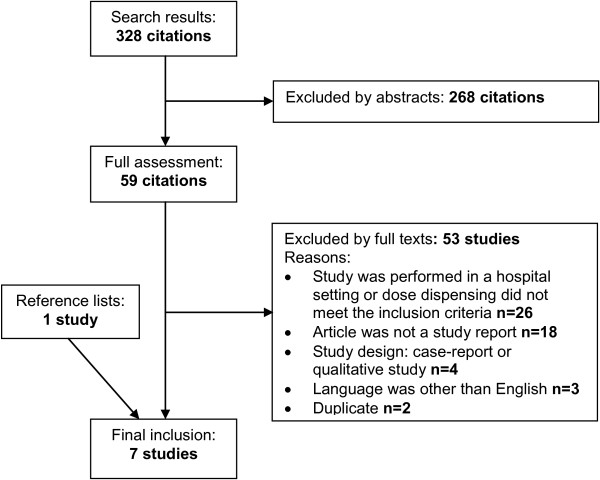
Flow chart of the study selection process.

Seven studies met the inclusion criteria (Figure 1) [15-21]. In all, 328 citations were found in the literature search. A total of 59 of the citations were retrieved as full texts and 53 of these were excluded. One of those included was found in the reference list of the included study. No relevant studies were found in the Finnish literature.

The data extraction of the studies selected is presented in Table 2. Six out of seven studies were conducted in the Nordic countries, [15-19,21] and one was from the Netherlands [20]. Five studies were register-based studies [15,16,18,19,21]. Other studies applied before-after, [17] and observational study designs [20]. Four of the studies had control group [15-18]. Two of the register-based studies were descriptive, cross-sectional studies without any follow-up of the ADD intervention [19,21]. Seven of the studies included a description of ADD [15-18,20,21], which varied between the studies. The quality assessment showed that the quality was good in four of the studies [16-18,20] and acceptable in three (Additional file 1) [15,19,21].

### Appropriateness of medication use

Appropriateness of medication use was investigated in five of the studies [15,16,18,19,21]. In four of these the focus was on potential inappropriate drug use [16,18,19,21], two of which were controlled. All of the studies were conducted in Sweden. Inappropriate drug use was measured, using the following quality indicators: use of long-acting benzodiazepines, use of anticholinergic drugs, use of three or more psychotropic drugs, drug duplications, use of 10 or more drugs, and potential drug-drug interactions [23].

#### Controlled studies (n = 3)

In the controlled studies, patients using the ADD scheme were those with higher prevalences of potential inappropriate drug use according to all quality indicators, than those using a standard dispensing procedure (5.9-55.1% *vs.* 2.4-4.9%) [16,18]. After controlling the results of the representative population-based register study for age and number of drugs dispensed, patients using the ADD scheme had a higher risk using anticholinergic and three or more psychotropic drugs (ORs 1.43-4.93; 95% CI 1.40-5.17) [18]. The contrasting association prevailed for long-acting benzodiazepine use among women and drug-drug interactions among women and men (ORs 0.69-0.80; 95% CI 0.66-0.83). When the results of the regional register study were adjusted for age, sex, burden of disease, and residence, the risk for inappropriate drug use was higher among the ADD users than in patients using a standard dispensing procedure, according to all indicators applied (ORs 1.36-5.48; 95% CI 1.18-6.30) [16]. In this study, more comprehensive controlling of the confounding factors was applied than in the population-based register study [18].

Drug treatment changes were studied in Sweden [15]. Drug treatment of the ADD users was more likely to remain unchanged than in patients using a standard dispensing procedure (OR 1.66, 95% CI 1.20-2.31, adjusted for age, sex, cognition, year of data collection, and subgroup of drug).

#### Uncontrolled studies (n = 2)

The prevalences of potential inappropriate drug use were higher among 65-79-year-old ADD users than older users (≥80 years) in the uncontrolled regional register studies (*n* = 2) [19,21].

### Medication safety

The influence of ADD on medication safety was investigated in two of the studies, of which one was controlled [17] and the other uncontrolled [20].

#### Controlled study (n = 1)

The controlled study conducted in Norway explored the impact of ADD on inconsistencies in medication records between general practitioners and home care services [17]. It showed that the ADD implementation reduced discrepancies in medication records by 34% (*P* < 0.001) between the general practitioners and home care services.

#### Uncontrolled study (n = 1)

The uncontrolled study investigated the frequency of medication administration errors and potential risk factors for these errors in nursing homes using ADD [20]. The study indicated that the risk of administration errors was higher when the medication was not supplied by ADD (OR 2.92; 95% CI 2.04-4.18).

### Costs

Costs were not investigated nor was economic evaluation performed in any of the studies.

## Discussion

Few studies have investigated the outcomes of the ADD service in primary healthcare, and the scientific evidence is too limited to draw any explicit conclusions on its effectiveness in improving the quality of pharmacotherapy. The findings of the controlled studies reviewed suggest that patients using the ADD service were those having more inappropriate drug use than the patients using the standard dispensing procedure. The findings also suggest that the ADD service may improve medication safety in terms of reducing discrepancies in the documentation of patient medication records in primary healthcare. At the same time, ADD may pose a risk of continuing the drug treatment unchanged for an unnecessarily long period if the medication is not regularly reviewed.

Two of the four controlled studies reviewed indicate that patients using the ADD scheme include those with more complicated drug regimens and high-risk medications, such as anticholinergics and psychotropics [16,18]. This finding is in line with the idea of ADD as a preventive intervention targeted to patients with a higher risk of drug-related problems, medication errors, or inappropriate drug use. There is recent evidence that ADD patients’ quality of pharmacotherapy may be improved by regular medication reviews integrated with ADD [24]. Medication reviews may also reduce the risk of unchanged medications for unnecessarily long periods, once a patient is enrolled in the ADD procedure [15]. These aspects support the idea that medication review should be integrated as a part of the ADD procedure to identify and solve inappropriate drug use. However, none of the seven studies included in the review indicated whether the standard ADD procedure applied involved a medication review to assure appropriateness of the dose-dispensed medications. In Finland, the Association of Finnish Pharmacies has recommended that each patient’s medications should be reviewed in the community pharmacy before they are enrolled in the ADD service [4]. The Ministry of Social Affairs and Health has recommended that medications for the elderly should be reviewed at least once per year [13]. Further research should be focused on this area to optimize the ADD procedure from the inappropriate drug use perspective.

The studies included applied quite a limited range of outcome measures. Outcome measures associated with costs were missing from all the studies. In future studies, it would be essential to estimate costs and benefits from different stakeholder points of view. These stakeholders include healthcare decision-makers and providers, patients and relatives, community pharmacies, and public insurance. When ADD systems are implemented in primary healthcare, it is also important to identify what kind of changes these systems make in nurses’ duties and allocation of working time, since they are mainly responsible for the distribution and administration of medicines to patients in home care services and nursing homes. Evidence from hospital settings indicates that changes in the work process can lead to new kinds of medication errors [25,26]. For example, nurses check the medicines less carefully because they rely on the automation. Therefore, it is important to involve parties of the medication process in the ADD implementation process. The work processes after ADD implementation of ADD should be assessed to ensure their safety in primary healthcare.

Even though evidence for the benefits of the ADD service in primary healthcare is limited, the service is officially implemented and widely used in the Nordic countries. This may be due to the urgent need for finding strategies and tools to ensure the safe use of medicines in a rapidly growing elderly population. Further research applying relevant study designs, methods, and outcome measures is needed to provide evidence for ADD service benefits in terms of medication safety, appropriateness of medication use, and costs.

### Strengths and limitations of the systematic review

So far, this is the first systematic review of ADD in primary healthcare. The literature search was performed in various databases with several keywords. Two researchers selected the studies independently. The Finnish literature was also searched.

The study has some limitations. The major limitation is the very limited published evidence for ADD in primary healthcare [27]. The seven studies that passed the inclusion criteria and reporting assessment had weaknesses in the study designs, sampling, and research methods, hindering the generalization of the findings. Three out of seven studies were uncontrolled [19-21], even though controlled studies provide more adequate evidence for the outcome of the intervention [28]. Only one of the studies was population-based [18]. Furthermore, the ADD service procedure varied between studies. The literature search was restricted to starting from the year 1995. However, in a narrative search done before the systematic search, studies from the late 1980s and early 1990s were not found, because the earliest time the ADD service was launched in primary healthcare was in the late 1980s in Sweden [5].

## Conclusions

A very limited number of controlled studies have explored ADD in primary healthcare. Consequently, the evidence for ADD’s influence on appropriateness and safety of medication use is limited, and lacking in information on costs. The findings of this review suggest that patients using the ADD have more inappropriate drugs in their regimens, and that ADD may improve medication safety in terms of reducing the discrepancies in medication records. Further evidence is needed to draw sound conclusions on ADD’s outcomes.

## Competing interests

The authors declare that they have no competing interests.

## Authors’ contributions

JS participated in the design of the study, carried out the selection of the studies, analyzed and interpreted the data, and drafted the manuscript. SS participated in the design of the study, carried out the selection of the studies, and revised the manuscript critically. JI participated in the design of the study, carried out the literature research, and revised the manuscript critically. MB participated in the design of the study and revised the manuscript critically. MA participated in the design of the study, interpreted the data, and revised the manuscript critically. AM participated in the design of the study, revised the manuscript critically, and provided supervision of the project. All authors read and approved the final manuscript.

## Supplementary Material

Additional file 1 Quality assessment of the selected studies with the STROBE checklist. Click here for file
